# Effect of pre- and postharvest treatments on the quality and storage ability of fresh artichoke heads: opinion article

**DOI:** 10.3389/fpls.2024.1368901

**Published:** 2024-02-16

**Authors:** Mohamed M. El-Mogy, Nahed M. Rashed, Saleh M. AlTurki, Tong Chen

**Affiliations:** ^1^ Department of Arid Land Agriculture, College of Agricultural and Food Science, King Faisal University, Al-Ahsa, Saudi Arabia; ^2^ Department of Vegetable Crops, Faculty of Agriculture, Cairo University, Giza, Egypt; ^3^ Horticulture Department, Faculty of Agriculture, Damietta University, Damietta, Egypt; ^4^ Key Laboratory of Plant Resources, Institute of Botany, Chinese Academy of Sciences, Beijing, China

**Keywords:** *Cynara cardunculus var*. *scolymus*, shelf-life, quality, storage, phenolic compounds, ready to eat

## 1 Introduction

Globe artichoke (*Cynara cardunculus* var. *scolymus*) is a perennial plant belonging to the *Asteraceae* family. The edible part of the globe artichoke plant (known as the head or bud) is the immature inflorescence before any sign of opening, the enlarged receptacle (bottom), as well as the tender bases of the bracts, which are used all over the world for fresh consumption or minimally processed (fresh-cut or ready to eat) (Pandino and Mauromicale, 2020). The best quality indexes for globe artichoke heads are free from discoloration, green, tightly closed bracts, tender, and free from any other defects (Mencarelli et al., 1993). After harvesting and during the handling processes and shipping, globe artichoke heads may lose their quality, which is represented by bracts wilting, opening, and yellowing (Ricci et al., 2013).

In 2021, according to FAOSTAT, the global production of globe artichoke was 1470332 tones, harvested from 116350 ha. Additionally, the value of globe artichoke exports globally reached 62851000 US $, while the value of imports globally reached 73259000 US $. The European Union is the largest producer (55.6% of total world production, according to FAOSTAT in 2021) and consumer of globe artichokes, with Italy, Spain, and Egypt being the major producers (FAOSTAT, 2021). Other significant globe artichoke producing regions include Africa, the Americas, and Asia, with 21.1%, 14.7%, and 8.7% of global production, respectively.

Additionally, the rising awareness of the health benefits of globe artichokes is also contributing to the market’s growth. It has been well known that globe artichoke contains several antioxidants and health compounds, including polyphenols (Dabbou et al., 2017; Iglesias-Carres et al., 2023), dairy fibers, flavonoids (Lattanzio et al., 2009; Pandino et al., 2012), and minerals (Lombardo et al., 2017a). Researchers have already found a link between heart disease and phenolic compounds, like caffeic acid and chlorogenic acid, which are found in globe artichoke (Li et al., 2020b). However, postharvest losses are a major concern for producers and consumers. Postharvest technology and treatments are essential to maintain the quality and extend the shelf life of globe artichoke. Moreover, minimally processed globe artichoke heads are more sensitive, deteriorate vastly and become more acceptable to microbial contamination (Ahvenainen, 1996). Several pre-harvest and post-harvest treatments can affect the quality and storage ability of globe artichokes. Thus, the objective of this review is to present the most recent postharvest technologies and treatments for globe artichoke.

## 2 Pre-harvest treatments

### 2.1 Mineral fertilization

Many previous studies demonstrated the correlation between mineral and/or organic fertilizers on the quality and postharvest behavior of horticulture crops (Kilic et al., 2021). It has been found that the balance of nitrogen, phosphor, and potassium fertilizers enhanced the sugar, ascorbic acid, and polyphenol content of globe artichoke (Lombardo et al., 2015a). For example, (Lombardo et al., 2017b) tested the effect of two levels of nitrogen fertilizer (200 kg ha^−1^ and 400 kg ha^−1^) on the quality of ready to eat globe artichoke heads during cold storage at 4°C for 12 days. The results showed that globe artichoke heads from plants that received nitrogen fertilizer had higher inulin contents and sensory levels than those from plants without nitrogen fertilizer. Additionally, during cold storage periods, lower mesophilic and psychotropic counts were observed in the heads obtained from plants that received nitrogen fertilizer compared to the control. The balance of minerals during fertilizer is important to ensure high production and an early yield of globe artichoke. In this regard, (Ierna et al., 2012) studied the effect of phosphor levels (50 and 150 kg P_2_O_5_ ha^−1^) and nitrogen levels (0, 150, 300 and 450 kg N ha^−1^) on the early yield, heads characteristic, and nutrient efficiency of two globe artichoke cultivars. The results show that 150 kg P_2_O_5_ ha^−1^ reduced the utilization of nitrogen fertilizer from 450 to 300 kg N ha^−1^ without reduction in the yield or effect on the early yield. In addition, increasing the nitrogen level from 0 to 150 kg N ha^−1^ increased the total yield (number of heads/hectare and the weight of main and secondary heads) by 48%, while the highest rate of nitrogen (450 kg N ha^−1^) didn’t have any extra effects. Nitrogen fertilizer could also affect the green color degree of globe artichoke. For example, it has been found that adding 200 Kg N per hectare increased the chlorophyll content of two globe artichoke cultivars, namely “Violet de Provence” and “Tema 2000”, compared to the non-fertilized plants (Lombardo et al., 2020). The effect of nitrogen fertilizer on the quality of globe artichoke was tested in previous works that concluded in **Table 1**. The results in **Table 1** show that the rate of optimal nitrogen level is varying and depends on several other factors. The impact of several pre-harvest factors, including fertilizers, on globe artichoke quality was mentioned in detail before (Lombardo et al., 2018).

**Table 1:** Influence of nitrogen fertilizer on the quality of globe artichoke.

Nitrogen treatment	Effect	References
200 and 400 kg N ha^−1^	Increase inulin contents and sensory level and decrease mesophilic and psychotropic counts	(Lombardo et al., 2017b)
150 kg N ha^−1^	Increased number of heads/hectare and the weight of main and secondary heads.	Ierna et al., 2012
200 kg N ha^−1^	Increased the chlorophyll content	Lombardo et al., 2020
200 kg N ha^−1^	Promote the earliness, increased the yield, and head weight.	(Paradiso et al., 2007)
300 kg N ha^−1^	Increase yield	(Ierna et al., 2006)
120 kg N ha^−1^	Increase marketable yield, head dry matter, and head size	(Shinohara et al., 2011)
100 kg N ha^−1^	Increase caffeoylquinic acids and luteolin	(Negro et al., 2013)

### 2.2 Oxalic acid

Oxalic acid has been used as a post-harvest treatment to extend the shelf-life of many crops, including globe artichoke (Ruíz-Jiménez et al., 2014). Additionally, when used as a pre-harvest treatment, oxalic acid has been shown to delay the postharvest ripening process and preserve the quality of some crops (Zhu et al., 2016). The effect of oxalic acid as a pre-harvest treatment to maintain quality and extend the shelf-life of globe artichoke during cold storage at 2°C for 21 days was evaluated by Martínez-Esplá et al. (Martínez-Esplá et al., 2017a). The results indicated that pre-harvest oxalic acid treatment increased the first class globe artichoke heads (less rates of opening of bracts, misshapes, and bruising) compared to the control. Additionally, the results show that oxalic acid treatment reduced the respiration rate, weight loss, and conserved total phenolics, hydroxycinnamics, and luteolins concentrations at harvest time and during refrigerated storage.

### 2.3 Gibberellins

The exogenous infusion of gibberellins speeds up and synchronises flowering and increases yield by stimulating cell elongation and division (Mauromicale and Ierna, 2000). To extend the shelf-life and quality of fresh fruits and vegetables, gibberellins have been used as a pre-harvest treatment to delay the degreening of okra fruits (Xiao et al., 2022) and the repining of tomato fruits (Li et al., 2019). The influence that the gibberellin treatment has on the quality of the crop after it has been harvested has received some attention. Since the head’s accelerated growth results in a loss in its final weight, an increase in the length of its bracts, and, in some situations, a deformation in its shape, its effect is generally negative (Basnizki, 2019). Additionally, gibberellin-treated globe artichoke heads have a tendency to contain more water than untreated ones do, and they are also more vulnerable to damage from low temperatures or disease attacks. However, the bioactive compounds in globe artichoke heads could be enhanced by gibberellins treatment. In this respect, it has been found that the polyphenol, cynaropicrin, and caffeoylquinic acid contents of the globe artichoke heads were enhanced after treatment with gibberellin (Lombardo et al., 2022). Additionally, gibberellins treatment was able to shorten the time to the first harvest. The timing of gibberellin treatment, cultivars of globe artichoke, and gibberellin concentrations are factors that affect the effectiveness (either positive or negative) of gibberellin (Elia et al., 1994; Goreta et al., 2004; Othman and Leskovar, 2022). A recent study (Othman and Leskovar, 2022) examined the impact of exogenous gibberellins treatment date (4th and 8th leaf stages) and frequency (2 and 3 times) on morphological and physiological parameters, yield, and head quality in a warm and semi-arid climate for globe artichoke. The results showed that gibberellins treatment frequency (two or three) did not affect head quality or yield. Foliar gibberellins application at the 8-leaf stage increased yield by 13%, chlorogenic acid by 19%, cynarin by 30%, and total N by 20% compared to the 4-leaf stage. Also, a previous study found that gibberellins treatment increased phenolic compounds in globe artichoke heads (Rotondo et al., 2022).

### 2.4 Methyl jasmonate

Jasmonic acid and its conjugate, methyl jasmonate, have the ability to regulate fruit ripening, pollen viability, root growth, and plant defense against insects and pathogen, which result in the synthesis of bioactive secondary compounds (Creelman and Mullet, 1997; Sirhindi et al., 2020). In results, methyl jasmonate was used as a pre-harvest treatment to enhance the bioactive compounds and quality of some cops (Castillo et al., 2015; Ozturk et al., 2015). However, the previous works that evaluated the effect of jasmonic acid or methyl jasmonate on globe artichoke were rare. For example, Martínez-Esplá et al. (Martínez-Esplá et al., 2017b) studied the effect of pre-harvest methyl jasmonate application on the yield, quality, phenolic content, and antioxidant activity of globe artichoke heads. The results showed that methyl jasmonate application increased the quality, phenolic content, hydroxycinnamic acids, antioxidant activity, and luteolin of heads either at harvest or during refrigerated storage at 2°C for 28 days. Further studies are required on this topic.

### 2.5 Harvest time

The timing of harvest determines the concentration of phytochemicals in globe artichoke, influencing the overall nutritional quality of this crop. In this regard, (Licciardello et al., 2017) studied the effect of harvest time on the chemical compositions of three globe artichoke cultivars (‘Apollo’, ‘Exploter’ and ‘Spinoso di Palermo’). The results showed that regardless of the cultivars, globe artichoke heads sliced from the early harvest had a shelf life of 11 days, while those from the late harvest had a shelf life of 7 days. In previous work (Pandino et al., 2013), to determine the impact of harvest time on the polyphenol content of globe artichokes, field-grown globe artichoke of the re-flowering cultivar ‘Violetto di Sicilia’ were harvested monthly from November to April. The results showed that early harvest during February increased the total polyphenol content in the leaves, floral stem, and bracts, while the total polyphenol content in the receptacle of globe artichoke heads increased during April.

## 3 Post-harvest treatments

Globe artichoke heads are distributed either whole or prepared for ready consumption. The handling and postharvest operations of whole globe artichoke heads are easier and less expensive than minimally processed globe artichoke. Adopting minimal processing steps, including washing, removing external leaves, slicing, and packaging, can offer significant benefits for the commercialization of artichokes. This approach helps cut down on transport costs, storage space requirements, and preparation time for consumers (Ahvenainen, 1996). Nevertheless, these procedures trigger enzymatic browning, leading to a decline in quality linked to factors such as water loss, softening, microbial contamination, increased respiration, and ethylene production. These factors collectively contribute to a diminished shelf life (Ghidelli et al., 2013). Thus, more attention and a new approach are required for extending the shelf-life of minimally processed globe artichoke. Here, we will discuss the most recent technology for this topic.

### 3.1 Modified atmosphere packaging

MAP involves modifying the composition of the air surrounding the products to reduce respiration rates and slow down the ripening process. The most common gases used in MAP are carbon dioxide (CO_2_) and nitrogen (N). The effect of MAP on the quality of globe artichoke varies and depends on different factors such as gas concentrations, globe artichoke cultivars, combined treatments with MAP, packaging materials, and storage conditions. For example, the use of MAP with low O_2_ (5–10 kPa) and/or elevated CO_2_ (5–18 kPa) levels had no effect on the general appearance of fresh-cut globe artichoke compared with the control (normal atmospheric conditions) (Gil-Izquierdo et al., 2002; Ghidelli et al., 2015). Furthermore, coating (soy protein plus beeswax) with modified atmospheres (80 kPa O_2_) did not extend the shelf-life of fresh-cut globe artichoke; however it maintained the product’s antioxidant capacity as compared to the control packaging conditions (Ghidelli et al., 2015). In another experiment (La Zazzera et al., 2015), globe artichoke head halves were stored at 4°C for 9 days in modified atmosphere packaging containing 5% O_2_ and 10% CO_2_ in four different materials or in air in macro-perforated bag, which served as a control. The four materials were polylactic acid, polylactic acid with a single micro-perforation line, polypropylene with two micro-perforation lines, and polypropylene + polyamide with two micro-perforation lines. The results showed that all treatments (without significant differences between them) conserved the green colour of heads and had better quality compared to the control. It has been found that the microbiological safety and quality of the fresh globe artichoke hearts, which were sliced, packaged in a modified atmosphere, and stored under cold conditions for 12-15 days, were ensured due to the absence of *E. coli, Salmonella*, *L. monocytogenes*, fecal coliforms, and sulfite-reducing bacteria, as well as the low levels of aerobic mesophilic bacteria, psychrotrophic microorganisms, Enterobacteriaceae, molds, and yeasts (García-Martínez et al., 2017).

### 3.2 Calcium chloride

The effective role of calcium chloride in slowing brown discoloration and prolonging the storage period has been proven in many previous studies (Manganaris et al., 2007; El-Mogy et al., 2020; Li et al., 2020a). Treatment with calcium chloride was used to extend the shelf life of globe artichokes. For example, fresh-cut globe artichokes coated with *Cordia myxa* gum and loaded with calcium chloride (1%) were then stored at 2°C for 9 days (El-Mogy et al., 2020). The results showed that edible coatings supplemented with calcium chloride significantly reduced weight loss and conserved vitamin C and phenolic compounds compared with *Cordia myxa* gum treatment without calcium chloride enrichment.

### 3.3 Edible coatings

Edible coatings are applied to the surface of the globe artichokes to reduce moisture loss and prevent decay. The most common coatings used are chitosan, alginate, carboxymethyl cellulose (CC), and whey protein. Carboxymethyl cellulose (CC) is an anionic polysaccharide and water-soluble (Gol et al., 2013). Previous works used CC as a fruit coating material to prolong the shelf-life of some crops (Koushesh Saba and Sogvar, 2016). Additionally, a soy protein plus beeswax edible coating and l-cysteine were the most effective treatments for controlling enzymatic browning and increasing the storage ability of fresh-cut globe artichokes without producing any off-odors (Ghidelli et al., 2015). In another study (Rizzo et al., 2019), the impact of two treatments on ‘Spinoso sardo’ ready to use globe artichoke slices during an 11-day storage period at 4°C was assessed. The treatments were (i) anti-browning treatments involving either citric acid (0.5%) + ascorbic acid (2%) or cysteine (0.5%, w/v), and (ii) immersion in locust bean gum edible coating, with or without *Foeniculum vulgare* essential oil. The results showed that locust bean gum edible coating with *Foeniculum vulgare* was the best treatment for maintaining sensory qualities, bioactive compounds, and physical parameters, as well as reducing microbial growth. Also, fresh-cut globe artichokes coated with *Cordia myxa* gum and loaded with calcium chloride (1%) significantly reduced weight loss and conserved vitamin C and phenolic compounds compared with the control treatment (El-Mogy et al., 2020).

### 3.4 Ozonated water and gaseous ozone

Ozone application, which has been used to disinfect many water resources, is one of the most promising technologies from the perspectives of health and the environment and has recently gained attention as a potential antibacterial agent for use in the post-harvest treatment of fruits and vegetables (Ozkan et al., 2011). In this regard, (Restuccia et al., 2014) studied the effects of the combination of ozonated water and ozone gas or its individual of them on the quality, microbial growth, and chemical compositions of two globe artichoke cultivars (Violet de Provence and Romanesco clone C3) during refrigerated storage at 4°C. The results showed that treatments were affected by the different cultivars. Violet de Provence cv. was more sensitive to ozone treatment (higher respiration rate and senescence rate) than cv. Romanesco clone C3. Additionally, ozone treatment reduced the microbial growth on the globe artichoke heads. In a previous study, the use of ozonized water for washing globe artichoke heads and storage under an O_3_-enriched atmosphere resulted in increased water retention, particularly noticeable for the Violet de Provence cultivar compared with Apollo, when compared to the control (Lombardo et al., 2015b). However, after being stored in an ozone-filled room for an extra four days, both the Apollo and Violet de Provence cultivars showed a noticeable drop in their antioxidant activity and total polyphenol content. Hence, the authors recommended that the duration of exposing globe artichoke heads to an ozone-enriched atmosphere should not exceed 3 days.

The impact of storing three globe artichoke cultivars (‘Violet de Provence,’ ‘Tema 2000,’ and ‘Apollo’), harvested at various times (winter, early spring, late spring), at 4°C under ozone-enriched atmospheres was investigated (Lombardo et al., 2015c). The artichokes were assessed after 0, 3, and 7 days of storage for changes in microbiological quality and antioxidant content.

The results show a notable decrease in mesophilic bacteria, as well as yeasts and molds, in artichoke heads stored in an ozone-enriched atmosphere for 3 or 7 days. The most substantial reductions were noted in the Tema 2000 and Apollo cultivars, which were harvested in winter and early spring, respectively. Additionally, in the winter harvest, ozone-treated heads of ‘Violet de Provence’ exhibited significant retention of ascorbic acid, while in the early spring harvest, ozone-treated heads of ‘Apollo’ displayed increased total polyphenol content.

### 3.5 Ascorbic acid and citric acid

Ascorbic acid (vitamin C) is known as an anti-browning agent for minimizing the browning of the surface of fresh fruits and vegetables during storage (Arnold and Gramza-Michałowska, 2022). Many factors, such as phenotypes, the concentration of ascorbic acid, and the concentration of phenolic compounds, affect the efficiency of ascorbic acid (Ghidelli et al., 2013). Globe artichoke cultivars vary in the concentration of phenolic compounds (Cabezas-Serrano et al., 2009). Previous works are emphasize the role of ascorbic acid in minimizing the browning of fresh-cut globe artichoke and enhancing its quality. For example, Lattanzio et al. (Lattanzio et al., 1989) studied the effect of dipping globe artichoke heads in ascorbic acid at a rate of 1% on the progress of the browning rate. The results showed that ascorbic acid treatment reduced the browning degree and enhanced the quality and shelf-life of globe artichoke heads that were kept in closed polyethylene bags at 4°C for 50 days. Also, Amodio et al. (Amodio et al., 2011) found that fresh-cut globe artichoke treated with 1% ascorbic acid had a higher appearance degree compared with the control. Other works mentioned that ascorbic acid and citric acid as anti-browning agents help maintain the quality and extend the shelf-life of fresh-cut globe artichoke when combined with other treatments such as certain packaging materials (Muratore et al., 2015) and coatings (El-Mogy et al., 2020). On the contrary, Ghidelli et al. (Ghidelli et al., 2013) found that postharvest ascorbic acid treatment at different rates (0.5%, 1%, 1.5%, or 2%) didn’t affect the browning degree of fresh-cut globe artichoke. Further work is required to study the effect of ascorbic acid on the quality and browning of globe artichokes.

### 3.6 Cysteine treatment

Previous work found that pH degrees (ranging from 2 to 7) affect the activity and effectiveness of l-cysteine hydrochloride monohydrate (5%) on the storage ability of fresh-cut globe artichoke, which was stored at 5°C for 12 days (Cabezas-Serrano et al., 2013). The results showed that the highest appearance and the lowest browning rate were observed when fresh-cut globe artichokes were treated with l-cysteine hydrochloride monohydrate at pH 7. The lowest PPO activity was observed at this pH degree. Also, (Amodio et al., 2011) found that postharvest treatment with cysteine (0.5%) was the most effective treatment for minimizing the progress of browning on the surface of fresh-cut globe artichoke compared to the control treatment and other treatments (ascorbic acid, citric acid, ethanol, sodium chloride, and 4-hexylresorcinol). In a previous study (Giménez et al., 2023), the impact of L-cysteine, both alone and in combination with a mixture of essential oil components (eugenol, thymol, and carvacrol), on the browning, quality, and bioactive compounds of fresh-cut artichokes during a 9-day storage period at 2°C was evaluated. The findings indicated that applying cysteine along with 150 µL of essential oils resulted in the least browning, highest antioxidant properties, and optimal quality and sensory parameters. This post-harvest treatment for fresh-cut globe artichokes could offer a natural and environmentally friendly solution to enhance their quality and extend their shelf life.

### 3.7 Packaging materials and essential oils

The type of packaging materials could affect the shelf-life and quality of fresh-cut globe artichoke (Ashraf et al., 2023). (Muratore et al., 2015) studied the effect of different packaging films (macroperforated, microperforated, and non-perforated) on the storage ability of two fresh-cut globe artichoke cultivars (cvs. ‘Violet de Provence’ and ‘Tema 2000’). The results showed that either non-perforated or microperforated films were effective for reducing moisture losses and enhancing globe artichoke storage ability compared with macro-perforated film. In addition, (Giménez et al., 2003) tested the effect of different five packing films (2 PVC and 3 P-Plus) on sensory quality and the growth of some microbes (mesophiles, psychrotrophs, anaerobic micro-organisms, sporeformers, faecal coliforms, *Salmonella* and *Escherichia coli*) in ready to eat fresh globe artichoke. The results indicated that, for the majority of treatments, there was no correlation observed between microbial growth and alterations in appearance. However, despite a rapid decline in sensory quality in treatments where the equilibrium atmosphere clearly displayed anaerobic conditions, microbial counts remained below the legally established microbiological limits.

The role of many essential oils in maintaining quality and extending the shelf-life of several crops has been approved (Sivakumar and Bautista-Baños, 2014). There is rare research discussing the effect of essential oils on the storage ability of globe artichoke heads. For example, Rizzo et al. (Rizzo et al., 2021) evaluated the effect of fennel essential oil on the fresh-cut globe artichoke during cold storage at 4°C for 12 days. The results showed that the globe artichoke cultivars differed in their responses to treatment with fennel essential oil. When fennel essential oil was used instead of the control treatment, the loss of firmness was less and the respiration rate, polyphenol oxidase activity, and microbial counts were lower.

### 3.8 Oxalic acid

The application of oxalic acid as a post-harvest treatment for increasing the shelf-life of horticulture crops was mentioned before in the literatures due to its role in decreasing respiration rate and ethylene production in climacteric fruits (Huang et al., 2013) and maintaining quality during cold storage of non-climacteric fruits (Valero et al., 2011). There is rare work on the effect of oxalic acid treatment on the quality and postharvest behaviour of globe artichokes. On this point, Ruíz-Jiménez et al. (Ruíz-Jiménez et al., 2014) studied the effect of dipping globe artichoke heads in 1 mM of oxalic acid solution for 10 minutes that was stored at 20°C for 3 days. The results indicated that oxalic acid treatment reduced weight loss, loss of firmness, discoloration, and microbial growth of globe artichoke heads, while total phenolics and antioxidant activity were not affected compared with the control treatment.

## 4 Conclusion

Globe artichokes offer health benefits such as antioxidants, health compounds, and minerals. However, postharvest losses pose a significant concern. Postharvest technologies are crucial to maintain quality, especially for minimally processed heads, which are prone to rapid deterioration and microbial contamination. Pre-harvest mineral fertilization enhances sugar, ascorbic acid, and polyphenol content. Oxalic acid, used for post-harvest treatment, delays ripening and preserves quality. Also, gibberellins as a pre-harvest treatment enhance bioactive compounds. Harvest timing influences phytochemical concentration, affecting nutritional quality of artichoke heads. Post-harvest treatments like washing, slicing, and packaging offer commercial benefits but can trigger browning, demanding innovative approaches for extended shelf-life. Thus, novel and promising postharvest technologies are required.

Many postharvest treatments were applied to extend the shelf-life of globe artichoke heads. Modified Atmosphere Packaging (MAP) reduces respiration rates and ripening. Calcium chloride slows browning, and edible coatings like chitosan and carboxymethyl cellulose extend shelf life. Ozone treatment reduces microbial growth but may decrease antioxidant activity. Ascorbic and citric acid reduce browning, with L-cysteine hydrochloride monohydrate and essential oils offering an environmentally friendly solution for fresh-cut artichokes. Packaging materials and essential oils impact shelf-life of globe artichoke. Also, non-perforated or micro-perforated films reduce moisture loss.

## Author contributions

ME-M: Data curation, Funding acquisition, Resources, Software, Validation, Visualization, Writing – original draft, Writing – review & editing. NR: Investigation, Methodology, Project administration, Writing – original draft. SAT: Formal analysis, Funding acquisition, Software, Supervision, Writing – review & editing. TC: Formal analysis, Methodology, Project administration, Software, Visualization, Writing – review & editing.

## References

[B1] Ahvenainen R. (1996). New approaches in improving the shelf life of minimally processed fruit and vegetables. Trends Food Sci. Technol. 7, 179–187. doi: 10.1016/0924-2244(96)10022-4

[B2] Amodio M. L. Cabezas-Serrano A. B. Peri G. Colelli G. (2011). Post-cutting quality changes of fresh-cut artichokes treated with different anti-browning agents as evaluated by image analysis. Postharvest Biol. Technol. 62, 213–220. doi: 10.1016/j.postharvbio.2011.05.004

[B3] Arnold M. Gramza-Michałowska A. (2022). Enzymatic browning in apple products and its inhibition treatments: A comprehensive review. Compr. Rev. Food Sci. Food Saf. 21, 5038–5076. doi: 10.1111/1541-4337.13059 36301625

[B4] Ashraf J. Z. Pati S. Fatchurrahman D. Amodio M. L. Colelli G. (2023). Screening of volatile organic compounds emitted from different packaging materials: case study on fresh-cut artichokes. Front. Sustain. Food Syst. 7. doi: 10.3389/fsufs.2023.1178104

[B5] Basnizki Y. (2019). Cynara scolymus, Handbook of flowering (Florida, USA: CRC Press), 391–399.

[B6] Cabezas-Serrano A. B. Amodio M. L. Colelli G. (2013). Effect of solution pH of cysteine-based pre-treatments to prevent browning of fresh-cut artichokes. Postharvest Biol. Technol. 75, 17–23. doi: 10.1016/j.postharvbio.2012.07.006

[B7] Cabezas-Serrano A. B. Amodio M. L. Cornacchia R. Rinaldi R. Colelli G. (2009). Screening quality and browning susceptibility of five artichoke cultivars for fresh-cut processing. J. Sci. Food Agric. 89, 2588–2594. doi: 10.1002/jsfa.3759

[B8] Castillo S. Valverde J. M. Guillén F. Zapata P. J. Díaz-Mula H. M. Valero D.. (2015). Methyl jasmonate and methyl salicylate affect differentially the postharvest ripening process of ‘primulat’ sweet cherry. 1079 ed (Leuven, Belgium: International Society for Horticultural Science (ISHS), 541–544.

[B9] Creelman R. A. Mullet J. E. (1997). Biosynthesis and action of Jasmonates in Plants. Annu. Rev. Plant Physiol. Plant Mol. Biol. 48, 355–381. doi: 10.1146/annurev.arplant.48.1.355 15012267

[B10] Dabbou S. Dabbou S. Flamini G. Peiretti P. G. Pandino G. Helal A. N. (2017). Biochemical characterization and antioxidant activities of the edible part of globe artichoke cultivars grown in Tunisia. Int. J. Food Properties 20, S810–S819. doi: 10.1080/10942912.2017.1315131

[B11] Elia A. Calabrese N. Bianco V. V. (1994). Sowing time, gibberellic acid treatments and cultivars of “seed” Propagated Artichoke. 371 ed (Leuven, Belgium: International Society for Horticultural Science (ISHS), 347–354.

[B12] El-Mogy M. M. Parmar A. Ali M. R. Abdel-Aziz M. E. Abdeldaym E. A. (2020). Improving postharvest storage of fresh artichoke bottoms by an edible coating of Cordia myxa gum. Postharvest Biol. Technol. 163, 111143. doi: 10.1016/j.postharvbio.2020.111143

[B13] FAOSTAT. (2021). Available at: https://www.fao.org/statistics/en/.

[B14] García-Martínez N. Andreo-Martínez P. Almela L. Guardiola L. Gabaldón J. A. (2017). Microbiological and sensory quality of fresh ready-to-eat artichoke hearts packaged under modified atmosphere. J. Food Prot. 80, 740–749. doi: 10.4315/0362-028X.JFP-16-289 28358262

[B15] Ghidelli C. Mateos M. Rojas-Argudo C. Pérez-Gago M. B. (2013). Antibrowning effect of antioxidants on extract, precipitate, and fresh-cut tissue of artichokes. LWT - Food Sci. Technol. 51, 462–468. doi: 10.1016/j.lwt.2012.12.009

[B16] Ghidelli C. Mateos M. Rojas-Argudo C. Pérez-Gago M. B. (2015). Novel approaches to control browning of fresh-cut artichoke: Effect of a soy protein-based coating and modified atmosphere packaging. Postharvest Biol. Technol. 99, 105–113. doi: 10.1016/j.postharvbio.2014.08.008

[B17] Gil-Izquierdo A. Conesa M. Ferreres F. Gil M. (2002). Influence of modified atmosphere packaging on quality, vitamin C and phenolic content of artichokes (Cynara scolymus L.). Eur. Food Res. Technol. 215, 21–27. doi: 10.1007/s00217-002-0492-3

[B18] Giménez M. J. Giménez-Berenguer M. Guillén F. Serna-Escolano V. Gutiérrez-Pozo M. Zapata P. J. (2023). Effect of cysteine with essential oils on quality attributes and functional properties of ‘Blanca de tudela’ Fresh-cut artichoke. Foods. doi: 10.3390/foods12244414 PMC10742624 38137218

[B19] Giménez M. Olarte C. Sanz S. Lomas C. Echávarri J. F. Ayala F. (2003). Relation between spoilage and microbiological quality in minimally processed artichoke packaged with different films. Food Microbiol. 20, 231–242. doi: 10.1016/S0740-0020(02)00146-6

[B20] Gol N. B. Patel P. R. Rao T. V. R. (2013). Improvement of quality and shelf-life of strawberries with edible coatings enriched with chitosan. Postharvest Biol. Technol. 85, 185–195. doi: 10.1016/j.postharvbio.2013.06.008

[B21] Goreta S. Bucan L. Dumicic G. (2004). Effect of environment and gibberellic acid (ga3) on earliness and yield of globe artichokes. 660 ed (Leuven, Belgium: International Society for Horticultural Science (ISHS), 155–159.

[B22] Huang H. Jing G. Guo L. Zhang D. Yang B. Duan X.. (2013). Effect of oxalic acid on ripening attributes of banana fruit during storage. Postharvest Biol. Technol. 84, 22–27. doi: 10.1016/j.postharvbio.2013.04.002

[B23] Ierna A. Mauro R. P. Mauromicale G. (2012). Improved yield and nutrient efficiency in two globe artichoke genotypes by balancing nitrogen and phosphorus supply. Agron. Sustain. Dev. 32, 773–780. doi: 10.1007/s13593-011-0048-7

[B24] Ierna A. Mauromicale G. Licandro P. (2006). Yield and harvest time of globe artichoke in relation to nitrogen and phosphorus fertilization. 700 ed (Leuven, Belgium: International Society for Horticultural Science (ISHS), 115–120.

[B25] Iglesias-Carres L. Bruno A. D’Antuono I. Linsalata V. Cardinali A. Neilson A. P. (2023). *In vitro* evidences of the globe artichoke antioxidant, cardioprotective and neuroprotective effects. J. Funct. Foods 107, 105674. doi: 10.1016/j.jff.2023.105674

[B26] Kilic N. Burgut A. Gündesli M. A. Nogay G. Ercisli S. Kafkas N. E.. (2021). The effect of organic, inorganic fertilizers and their combinations on fruit quality parameters in strawberry. Horticulturae. doi: 10.3390/horticulturae7100354

[B27] Koushesh Saba M. Sogvar O. B. (2016). Combination of carboxymethyl cellulose-based coatings with calcium and ascorbic acid impacts in browning and quality of fresh-cut apples. LWT - Food Sci. Technol. 66, 165–171. doi: 10.1016/j.lwt.2015.10.022

[B28] Lattanzio V. Kroon P. A. Linsalata V. Cardinali A. (2009). Globe artichoke: A functional food and source of nutraceutical ingredients. J. Funct. Foods 1, 131–144. doi: 10.1016/j.jff.2009.01.002

[B29] Lattanzio V. Linsalata V. Palmieri S. Van Sumere C. F. (1989). The beneficial effect of citric and ascorbic acid on the phenolic browning reaction in stored artichoke (Cynara scolymus L.) heads. Food Chem. 33, 93–106. doi: 10.1016/0308-8146(89)90112-X

[B30] La Zazzera M. Amodio M. L. Colelli G. (2015). Designing a modified atmosphere packaging (MAP) for fresh-cut artichokes. Adv. Hortic. Sci. 29, 24–29.

[B31] Li L. Su C. Chen X. Wang Q. Jiao W. Luo H.. (2020b). Chlorogenic acids in cardiovascular disease: A review of dietary consumption, pharmacology, and pharmacokinetics. J. Agric. Food Chem. 68, 6464–6484. doi: 10.1021/acs.jafc.0c01554 32441927

[B32] Li H. Wu H. Qi Q. Li H. Li Z. Chen S.. (2019). Gibberellins play a role in regulating tomato fruit ripening. Plant Cell Physiol. 60, 1619–1629. doi: 10.1093/pcp/pcz069 31073591

[B33] Li J. Zhou Q. Zhou X. Wei B. Zhao Y. Ji S. (2020a). Calcium treatment alleviates pericarp browning of ‘Nanguo’ Pears by regulating the GABA shunt after cold storage. Front. Plant Sci. 11. doi: 10.3389/fpls.2020.580986 PMC7522215 33042193

[B34] Licciardello F. Pandino G. Barbagallo R. N. Lombardo S. Restuccia C. Muratore G.. (2017). Quality traits of ready-to-use globe artichoke slices as affected by genotype, harvest time and storage time. Part II: Physiological, microbiological and sensory aspects. LWT - Food Sci. Technol. 79, 554–560. doi: 10.1016/j.lwt.2016.11.003

[B35] Lombardo S. Pandino G. Mauro R. P. Litrico A. Mauromicale G. (2020). Nitrogen efficiency and yield responses of two ‘early’ globe artichoke cultivars to nitrogen fertilization. 1284 ed (Leuven, Belgium: International Society for Horticultural Science (ISHS), 117–124.

[B36] Lombardo S. Pandino G. Mauromicale G. (2015a). The nutraceutical response of two globe artichoke cultivars to contrasting NPK fertilizer regimes. Food Res. Int. (Ottawa Ont.) 76, 852–859. doi: 10.1016/j.foodres.2015.07.042 28455071

[B37] Lombardo S. Pandino G. Mauromicale G. (2017a). Minerals profile of two globe artichoke cultivars as affected by NPK fertilizer regimes. Food Res. Int. 100, 95–99. doi: 10.1016/j.foodres.2017.08.028 28888464

[B38] Lombardo S. Pandino G. Mauromicale G. (2018). The influence of pre-harvest factors on the quality of globe artichoke. Sci. Hortic. 233, 479–490. doi: 10.1016/j.scienta.2017.12.036

[B39] Lombardo S. Pandino G. Restuccia C. Muratore G. Licciardello F. Mauro R. P.. (2015b). Effect of cultivar x ozone treatment interaction on the total polyphenols content and antioxidant activity of globe artichoke. Ital. J. Agron. 10, 105–107. doi: 10.4081/ija.2015.634

[B40] Lombardo S. Restuccia C. Muratore G. Barbagallo R. N. Licciardello F. Pandino G.. (2017b). Effect of nitrogen fertilisation on the overall quality of minimally processed globe artichoke heads. J. Sci. Food Agric. 97, 650–658. doi: 10.1002/jsfa.7784 27144811

[B41] Lombardo S. Restuccia C. Pandino G. Licciardello F. Muratore G. Mauromicale G. (2015c). Influence of an O3-atmosphere storage on microbial growth and antioxidant contents of globe artichoke as affected by genotype and harvest time. Innovative Food Sci. Emerg. Technol. 27, 121–128. doi: 10.1016/j.ifset.2014.12.007

[B42] Lombardo S. Scavo A. Pandino G. Cantone M. Mauromicale G. (2022). Improvement in the cynaropicrin, caffeoylquinic acid and flavonoid content of globe artichokes with gibberellic acid treatment. Plants. doi: 10.3390/plants11141845 PMC9324294 35890480

[B43] Manganaris G. A. Vasilakakis M. Diamantidis G. Mignani I. (2007). The effect of postharvest calcium application on tissue calcium concentration, quality attributes, incidence of flesh browning and cell wall physicochemical aspects of peach fruits. Food Chem. 100, 1385–1392. doi: 10.1016/j.foodchem.2005.11.036

[B44] Martínez-Esplá A. García-Pastor M. E. Zapata P. J. Guillén F. Serrano M. Valero D.. (2017a). Preharvest application of oxalic acid improves quality and phytochemical content of artichoke (Cynara scolymus L.) at harvest and during storage. Food Chem. 230, 343–349. doi: 10.1016/j.foodchem.2017.03.051 28407920

[B45] Martínez-Esplá A. Valero D. Martínez-Romero D. Castillo S. Giménez M. J. García-Pastor M. E.. (2017b). Preharvest application of methyl jasmonate as an elicitor improves the yield and phenolic content of artichoke. J. Agric. Food Chem. 65, 9247–9254. doi: 10.1021/acs.jafc.7b03447 28960971

[B46] Mauromicale G. Ierna A. (2000). Characteristics of heads of seed-grown globe artichoke [Cynara cardunculus L. var. scolymus (L.) Fiori] as affected by harvest period, sowing date and gibberellic acid. Agronomy 20, 197–204. doi: 10.1051/agro:2000119

[B47] Mencarelli F. Massantini R. Casella M. (1993). The influence of chemicals, stem length and plastic films on the quality of artichoke buds. J. Hortic. Sci. 68, 597–603. doi: 10.1080/00221589.1993.11516390

[B48] Muratore G. Restuccia C. Licciardello F. Lombardo S. Pandino G. Mauromicale G. (2015). Effect of packaging film and antibrowning solution on quality maintenance of minimally processed globe artichoke heads. Innovative Food Sci. Emerg. Technol. 31, 97–104. doi: 10.1016/j.ifset.2015.06.010

[B49] Negro D. Montesano V. De Lisi A. Mastro M. A. Rubino P. Sonnante G.. (2013). Productive and nutraceutical effects of globe artichoke fertilization. 983 ed (Leuven, Belgium: International Society for Horticultural Science (ISHS), 283–288.

[B50] Othman Y. A. Leskovar D. I. (2022). Foliar application of gibberellic acid improves yield and head phenolic compounds in globe artichoke. Sci. Hortic. 301, 111115. doi: 10.1016/j.scienta.2022.111115

[B51] Ozkan R. Smilanick J. L. Karabulut O. A. (2011). Toxicity of ozone gas to conidia of Penicillium digitatum, Penicillium italicum, and Botrytis cinerea and control of gray mold on table grapes. Postharvest Biol. Technol. 60, 47–51. doi: 10.1016/j.postharvbio.2010.12.004

[B52] Ozturk B. Yıldız K. Ozkan Y. (2015). Effects of pre-harvest methyl jasmonate treatments on bioactive compounds and peel color development of “Fuji” Apples. Int. J. Food Properties 18, 954–962. doi: 10.1080/10942912.2014.911312

[B53] Pandino G. Lombardo S. Lo Monaco A. Mauromicale G. (2013). Choice of time of harvest influences the polyphenol profile of globe artichoke. J. Funct. Foods 5, 1822–1828. doi: 10.1016/j.jff.2013.09.001

[B54] Pandino G. Lombardo S. Mauromicale G. Williamson G. (2012). Characterization of phenolic acids and flavonoids in leaves, stems, bracts and edible parts of globe artichokes. 942 ed (Leuven, Belgium: International Society for Horticultural Science (ISHS), 413–417.

[B55] Pandino G. Mauromicale G. (2020). Globe artichoke and cardoon forms between traditional and modern uses. 1284 ed (Leuven, Belgium: International Society for Horticultural Science (ISHS), 1–18.

[B56] Paradiso R. Cuocolo B. De Pascale S. (2007). Gibberellic acid and nitrogen rate affect yield and quality of artichoke. Acta Hortic. 730, 211–216. doi: 10.17660/ActaHortic.2007.730.26

[B57] Restuccia C. Lombardo S. Pandino G. Licciardello F. Muratore G. Mauromicale G. (2014). An innovative combined water ozonisation/O3-atmosphere storage for preserving the overall quality of two globe artichoke cultivars. Innovative Food Sci. Emerg. Technol. 21, 82–89. doi: 10.1016/j.ifset.2013.10.009

[B58] Ricci I. Amodio M. L. Colelli G. (2013). Influence of pre-cutting operations on quality of fresh-cut artichokes (Cynara scolymus L.): Effect of storage time and temperature before cutting. Postharvest Biol. Technol. 85, 124–131. doi: 10.1016/j.postharvbio.2013.05.011

[B59] Rizzo V. Lombardo S. Pandino G. Barbagallo R. N. Mazzaglia A. Restuccia C.. (2019). Shelf-life study of ready-to-cook slices of globe artichoke ‘Spinoso sardo’: effects of anti-browning solutions and edible coating enriched with Foeniculum vulgare essential oil. J. Sci. Food Agric. 99, 5219–5228. doi: 10.1002/jsfa.9775 31049967

[B60] Rizzo V. Lombardo S. Pandino G. Barbagallo R. N. Mazzaglia A. Restuccia C.. (2021). Active packaging-releasing system with foeniculum vulgare essential oil for the quality preservation of ready-to-cook (RTC) globe artichoke slices. Foods. doi: 10.3390/foods10030517 PMC8001857 33801354

[B61] Rotondo R. Rodríguez G. R. Escalante A. M. (2022). Gibberellin treatments increase the global performance of globe artichoke hybrid propagated by seeds and suckers. Sci. Hortic. 305, 111420. doi: 10.1016/j.scienta.2022.111420

[B62] Ruíz-Jiménez J. M. Zapata P. J. Serrano M. Valero D. Martínez-Romero D. Castillo S.. (2014). Effect of oxalic acid on quality attributes of artichokes stored at ambient temperature. Postharvest Biol. Technol. 95, 60–63. doi: 10.1016/j.postharvbio.2014.03.015

[B63] Shinohara T. Agehara S. Yoo K. S. Leskovar D. I. (2011). Irrigation and nitrogen management of artichoke: yield, head quality, and phenolic content. HortSci. horts 46, 377–386. doi: 10.21273/HORTSCI.46.3.377

[B64] Sirhindi G. Mushtaq R. Gill S. S. Sharma P. Abd_Allah E. F. Ahmad P. (2020). Jasmonic acid and methyl jasmonate modulate growth, photosynthetic activity and expression of photosystem II subunit genes in Brassica oleracea L. Sci. Rep. 10, 9322. doi: 10.1038/s41598-020-65309-1 32518304 PMC7283480

[B65] Sivakumar D. Bautista-Baños S. (2014). A review on the use of essential oils for postharvest decay control and maintenance of fruit quality during storage. Crop Prot. 64, 27–37. doi: 10.1016/j.cropro.2014.05.012

[B66] Valero D. Díaz-Mula H. M. Zapata P. J. Castillo S. Guillén F. Martínez-Romero D.. (2011). Postharvest treatments with salicylic acid, acetylsalicylic acid or oxalic acid delayed ripening and enhanced bioactive compounds and antioxidant capacity in sweet cherry. J. Agric. Food Chem. 59, 5483–5489. doi: 10.1021/jf200873j 21506518

[B67] Xiao X. Yang M. Dong W. Zhou C. Shi L. Chen W.. (2022). Gibberellic acid inhibited chlorophyll degradation in post-harvest okras. Postharvest Biol. Technol. 190, 111951. doi: 10.1016/j.postharvbio.2022.111951

[B68] Zhu Y. Yu J. Brecht J. K. Jiang T. Zheng X. (2016). Pre-harvest application of oxalic acid increases quality and resistance to Penicillium expansum in kiwifruit during postharvest storage. Food Chem. 190, 537–543. doi: 10.1016/j.foodchem.2015.06.001 26213007

